# The superior value of radiomics to sonographic assessment for ultrasound-based evaluation of extrathyroidal extension in papillary thyroid carcinoma: a retrospective study

**DOI:** 10.2478/raon-2024-0040

**Published:** 2024-09-15

**Authors:** Hui Zhu, Hongxia Luo, Yanyan Li, Yuhua Zhang, Zhijing Wu, Yan Yang

**Affiliations:** Department of Ultrasound, the Second Affiliated Hospital of Wenzhou Medical University, Wenzhou, China; Department of Statistical Science, University College London, London, United Kingdom

**Keywords:** ultrasonic radiomics, extrathyroidal extension, sonography, papillary thyroid carcinoma

## Abstract

**Background:**

Extrathyroidal extension was related with worse survival for patients with papillary thyroid carcinoma. For its preoperative evaluation, we measured and compared the predicting value of sonographic method and ultrasonic radiomics method in nodules of papillary thyroid carcinoma.

**Patients and methods:**

Data from 337 nodules were included and divided into training group and validation group. For ultrasonic radiomics method, a best model was constructed based on clinical characteristics and ultrasonic radiomic features. The predicting value was calculated then. For sonographic method, the results were calculated using all samples.

**Results:**

For ultrasonic radiomics method, we constructed 9 models and selected the extreme gradient boosting model for its highest accuracy (0.77) and area under curve (0.813) in validation group. The accuracy and area under curve of sonographic method was 0.70 and 0.569. Meanwhile. We found that the top-6 important features of xgboost model included no clinical characteristics, all of whom were high-dimensional radiomic features.

**Conclusions:**

The study showed the superior value of ultrasonic radiomics method to sonographic method for preoperative detection of extrathyroidal extension in papillary thyroid carcinoma. Furthermore, high-dimensional radiomic features were more important than clinical characteristics.

## Introduction

Thyroid cancer was one of the most pervasive carcinomas in clinic worldwide. From 1990 to 2017, its incident cases and deaths increased by 169% and 87%, respectively. China ranked the top 3 incident cases and South Asia had the largest number of deaths around the world in 2017.^1^ Papillary thyroid carcinoma (PTC) was the most common histological type of thyroid malignancy.^2^ Although it presented with indolent behavior, some patients still suffered from high risk of recurrence or death.^3,4^ Literatures showed that risk factors like male sex, older age, larger tumors size and extrathyroidal extension (ETE) could lead to more advanced disease.^4,5^ Besides, patients with ETE were reported to have worse survival and poorer prognoses than those without, no matter minimal ETE or gross ETE.^5,6^ In clinical practice, total/subtotal thyroidectomy was suggested for patients with ETE while hemithyroidectomy was recommended for those without.^7^ In order to avoid total/subtotal thyroidectomy in patients without ETE, a noninvasive way to preoperatively evaluate ETE was in urgent need.

ETE was defined as tumor extension of perithyroidal structures, which can be divided into minimal ETE (identified by histological examination) and gross ETE (identified by preoperative or intraoperative evidence) according to American Joint Committee on Cancer (AJCC) tumor-node-metastasis staging system.^8,9^ Preoperative ultrasonic (US) examination was the first-line diagnostic tool in detecting PTC.^10^ Several studies had revealed the predicting value of sonographic assessment for ETE. The reported sensitivity of sonographic assessment for ETE varied from 65.2% to 85.3%. And its specificity varied from 68.9% to 81.8%.^11,12,13^ Meanwhile, gross ETE (78%, 99.7%) was proven to have higher sensitivity and specificity than minimal ETE (30%, 93%).^10^ With such low diagnostic performance, more accurate method was in demand for ETE evaluation.

Radiomics analysis quantitatively extracted high-throughput features from medical images and converted them into mineable data to help diagnosing or predicting diseases in clinical practice.^14,15^ It combined radiology and machine learning and had been widely used in diagnosing disease and prognosing outcome.^2,16,17^ Recent studies had shown that radiomic features of magnetic resonance imaging (MRI), computer tomography (CT) and US images of PTC had potential predicting value in ETE with high area under the curve (AUC) of 0.812–0.906.^7,8,15,18^ However, little was known about the superior predicting value of US radiomics method to sonographic method.

The purpose of this study was to compare the predicting value of sonographic method and radiomics method of ETE based on US radiomic features and clinical characteristics of PTC nodules.

## Patients and methods

The study was conducted in accordance with the Declaration of Helsinki (as revised in 2013). The study was approved by institutional ethics board of the Second Affiliated Hospital of Wenzhou Medical University (NO.: 2021-K-20-01) and individual consent for this retrospective analysis was waived.

### Patients and clinical characteristics

This study included 460 consecutive patients (575 nodules) who were diagnosed with PTC pathologically from January 2018 to September 2019 in our hospital. The inclusion criteria were as follows: 1) preoperative US examination within two weeks of surgery; 2) initial surgical resection (total thyroidectomy, near total thyroidectomy or hemithy-roidectomy) with cervical dissection, and the ETE state was confirmed pathologically and/or by intraoperative evidence. The exclusion criteria were as follows: 1) incomplete clinical characteristics; 2) multifocal lesions in the same lobe of thyroid; 3) radiofrequency ablation before surgery. Finally, 310 patients (337 nodules) with mean age of 45.95 ± 11.69 years (from 12 to 77 years) and male-to-female ratio of 0.40 (89:221) were included in this study. Among them, 27 patients had one lesion on each lobe of the thyroid. According to previous study, minimal ETE was defined as an extension into the sternothyroid muscle or parathyroid soft tissues.^9^ And it was confirmed pathologically in this study. Meanwhile, gross ETE was defined as cancer invasion into the subcutaneous soft tissue, trachea, larynx, esophagus or recurrent laryngeal nerve.^9^ It was determined during surgery and further confirmed pathologically. Thus, nodules were divided into those with minimal ETE (n = 99), those with gross ETE (n = 4) and those without ETE (n = 234) (ETE negative group). Because of the very small sample of gross ETE, nodules with minimal ETE and gross ETE were categorized as ETE positive group.

**Figure 1.**
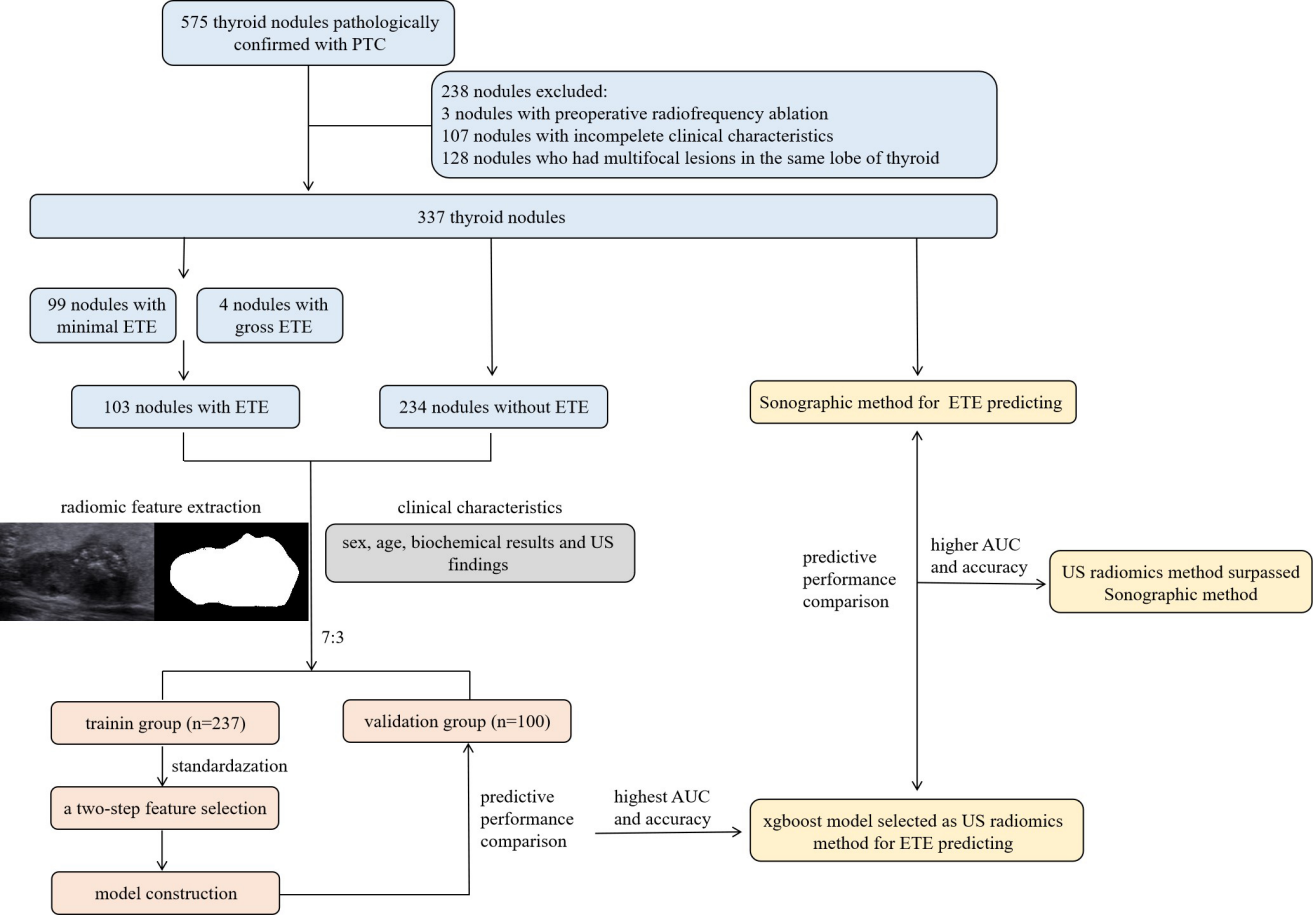
The flowchart for this retrospective study. AUC = area under the curve; ETE = extrathyroidal extension; PTC = papillary thyroid carcinoma; US = ultrasonic; xgboost = the extreme gradient boosting

Clinical characteristics comprised of age, sex, biochemical results and US findings. Standardized biochemical examination included those concerning preoperative assessment and those concerning thyroid function. Liver function analysis, renal function analysis, differential blood count and routine urianlysis were classified into former ones. Blood calcium ion, thyroid function analysis (free triiodothyronine 3 and 4, total triiodothyronine 3 and 4 and thyroid stimulating hormone) (FT3, FT4, TT3, TT4 and TSH), thyroglobulin (TG), antithyroglobulin antibodies (ANTITGAB) and antithyroid peroxidase antibody (ANTITPOAB) were classified into latter ones. The composition, echogenicity, shape, margin and echogenic foci consisted of the US findings of each nodule. They were measured, scored and classified according to the Thyroid Imaging Reporting and Data System (TI-RADS) criteria of American College of Radiology.^19^ A total of 34 clinical characteristics were enrolled. The flowchart of this study was shown in Figure 1.

### Sonographic method for extrathyroidal extension predicting

The assessment of ETE was performed by two US experts (YY, HL) who had 24 and 16 years of experience in US thyroid examination. Consensus were made when disagreements appeared and each of them reviewed half of the nodules without knowing the pathological state of ETE. Based on previous studies, sonographic ETE was suspected on US images when a capsular abutment or protrusion presented.^13^ Capsular abutment referred to nodules' contact of the thyroid capsule, which could be graded by their perimeter ratio (< 25%, 25–50% or > 50%). While protrusion referred to nodules protruding thyroid capsule and invading the muscles or soft tissue around the thyroid gland, including the trachea, fat space between the trachea, esophagus, esophagus sulcus, cervical sheath vessels, et al.^10,13^ The predictive performance of sonographic method was calculated using all nodules and compared by the AUC of the receiver operating characteristic (ROC), sensitivity, specificity, accuracy, positive predictive value (PPV) and negative predictive value (NPV).

### US radiomic feature extraction

Different US systems were employed in nodules' examination, including Esaote MyLab Class C (Esaote, Italy), Siemens ACUSON OXANA 2 (Siemens Medical Solutions, USA), GE Volume E8 (GE Medical Systems, USA), Hitachi HI VISION Preirus (Hitachi-Aloka Medical, Japan) and Mindray Resona 7T (Mindray Medical International, China). High-frequency linear probes (5 MHz to 14 MHz) were used depending on US systems. In order to have the best image for nodule, the depth and width of US systems were adjusted individually. Images of transverse and longitudinal section of nodules were saved as JPG files before the largest cross section of nodules were chosen for feature extraction. The US experts who were blinded to the pathological state of ETE reviewed the images, measured the US findings of nodules and delineated the region of interest (ROI) manually by Paint Win10. Consensus were made when disagreements appeared. Each expert review half of the nodules, respectively.

From those ROIs of US images, a total of 1769 US radiomic features were extracted using PyRadiomics (version 3.7, http://pyradiomics.readthedocs.io/en/latest/index.html). The features included 9 shape features, 360 first-order statistical features and 1400 textural features. Gray-level size-zone matrix (GLSZM), gray-level co-occurrence matrix (GLCM), gray-level dependence matrix (GLDM) and gray-level runlength matrix (GLRLM) consisted of the gray matrices in textural features. What's more, high-dimensional features were acquired by filters including Laplacian of Gaussian (LOG) with different sigma values (1.0 mm–10.0 mm with step 1.0 mm), square root, square, exponential, wavelet with 2D transform (low-pass/high-pass, LH; low-pass/low-pass, LL; high-pass/high-pass, HH; high-pass/low-pass, HL;), gradient and logarithm.

### US radiomics method for extrathyroidal extension predicting and comparison with sonographic method

All nodules were randomly assigned to training group (237 nodules) and validation group (100 nodules) by a ratio of 7:3. In order to show the randomness of grouping, the clinical characteristics between training group and validation group were compared. Then all features were enrolled in the following progression, including 1769 US radiomic features and 34 clinical characteristics. To eliminate redundant and irrelevant features as well as reduce variables recruited into model, a two-step features selection method was applied after standardization. Firstly, the minimum redundancy maximum relevance (mRMR) was used to select the most relevant features. Secondly, they were further selected by least absolute shrinkage and selection operator (LASSO) algorithm and candidate features were obtained. Ten-fold cross validation was used to avoid overfitting.^20^

In total, nine predicting models were constructed using these candidate features, including k nearest neighbors (KNN), binary logistics regression (LR), support vector machine (SVM), naivebayes (NB), randomforest (RF), decision tree (DT), adaptive boosting (adaboost), the extreme gradient boosting (xgboost) and gradient boosting machine (GBM). Their predictive performances were compared by AUC, sensitivity, specificity, accuracy, PPV and NPV. And the model with the highest AUC and accuracy was chosen as US radiomics method. Finally, the predictive performances of US radiomics method and sonographic method were compared. The statistical process was shown in Figure 1.

### Statistical analysis

Continuous variables were presented as mean ± standard deviation (SD) and were measured by Mann-Whitney U test or Student's t-test depending on the results of normality analysis. Categorical variables were presented as number (frequency) and Chi-square analysis or Fisher exact test were performed afterwards. SPSS software (version 19.0, IBM) was employed for the calculation mentioned above. The random allocation, standardization, mRMR, LASSO algorithm, KNN, binary LR, SVM, NB, RF, DT, adaboost, xgboost, GBM, DeLong's test and other statistical analysis were carried out with R software (version 4.0.3, MathSoft, http://www.r-project.org). DeLong's test was employed for statistical significance in AUC comparison.^21^ A value of *p* < 0.05 was considered statistically significant.

## Results

### Clinical characteristics of nodules

Clinical characteristics of training group and validation group were summarized in Table 1, including age, sex, biochemical results and US findings. The training group comprised of 237 nodules with a positive ETE rate of 30.38%. And the validation group comprised of 100 nodules with similar positive ETE rate of 31.00%. No significant differences were found in any characteristics between the two groups. Of all the nodules, images acquired by Esaote, Siemens, GE, Hitachi and Mindray systems accounted for 70.33% (237/337), 15.13% (51/337), 8.90% (30/337), 4.15% (14/337) and 1.48% (5/337).

**Table 1.** Characteristics of nodules in training and validation groups

**Characteristics**	**Training group (n = 237)**	**Validation group (n = 100)**	***p*-value**
Sex			0.708
Male	64 (27.00)	29 (29.00)	
Female	173 (73.00)	71 (71.00)	
Age (years)^a^	45.97 ± 11.92	46.70 ± 11.64	0.606
Size (mm)	9.69 ± 6.08	10.62 ± 7.60	0.550
WBC (×10^9/L)	5.96 ± 1.52	6.12 ± 1.45	0.241
NEUT (×10^9/L)	3.65 ± 1.26	3.75 ± 1.18	0.326
LYM (×10^9/L)	1.89 ± 0.56	1.91 ± 0.62	0.716
HB (g/L)	138.39 ± 16.01	141.70 ± 15.13	0.094
RBC (×10^12/L)	4.69 ± 0.46	4.70 ± 0.45	0.752
PLT (×10^9/L)	252.85 ± 58.65	255.70 ± 64.90	0.656
ALT (U/L)	23.50 ± 19.98	24.15 ± 18.41	0.280
AST (U/L)	21.53 ± 7.86	21.48 ± 7.68	0.696
ALB (g/L)	45.15 ± 3.19	44.93 ± 3.01	0.473
BUN (mmol/L)	4.83 ± 1.27	4.93 ± 1.29	0.391
CREA (umol/L)	58.14 ± 13.36	57.54 ± 12.82	0.489
UA (umol/L)	315.27 ± 82.01	325.80 ± 86.12	0.261
Ca (mmol/L)	2.40 ± 0.11	2.41 ± 0.10	0.460
TT3 (ng/ml)	1.10 ± 0.31	1.09 ± 0.18	0.828
TT4 (μg/dl)	8.32 ± 1.72	8.09 ± 1.62	0.329
FT3 (pg/ml)	3.36 ± 1.16	3.33 ± 0.41	0.346
FT4 (ng/dl)	1.30 ± 0.26	1.29 ± 0.20	0.829
TSH (μIU/ml)	1.66 ± 0.99	1.96 ± 1.38	0.090
ANTITGAB (IU/ml)	122.12 ± 325.06	114.13 ± 349.18	0.335
ANTITPOAB (IU/ml)	41.11 ± 101.64	52.08 ± 124.91	0.912
TG (ng/ml)	36.10 ± 72.10	42.00 ± 84.61	0.886
Urinary leukocyte^b^			0.412
Negative	188 (79.32)	87 (87.00)	
Positive 1+	19 (8.02)	5 (5.00)	
Positive 2+	17 (7.17)	3 (3.00)	
Positive 3+	10 (4.22)	3 (3.00)	
Positive 4+	10 (4.22)	2 (2.00)	
URBC^b^			0.144
Negative	208 (87.76)	89 (89.00)	
Positive 1+	21 (8.86)	6 (6.00)	
Positive 2+	6 (2.53)	1 (1.00)	
Positive 3+	0 (0)	2 (2.00)	
Positive 4+	2 (0.84)	2 (2.00)	
Urinary protein^b^			0.524
Negative	145 (61.18)	64 (64.00)	
Positive 1+	63 (26.58)	28 (28.00)	
Positive 2+	29 (12.24)	8 (8.00)	
Composition^b^			1.000
Predominately cystic	2 (0.84)	0 (0)	
Predominately solid	235 (99.16)	100 (100.00)	
Solid	0 (0)	0 (0)	
Echogenicity^b^			0.966
Hyperechoic or isoechoic	8 (3.38)	3 (3.00)	
Hypoechoic	191 (80.59)	82 (82.00)	
Markedly hypoechoic	38 (16.03)	15 (15.00)	
Shape^b^			0.974
Wider-than-tall	100 (42.19)	42 (42.00)	
Taller-than-wide	137 (57.81)	58 (58.00)	
Margin^b^			0.083
Smooth or ill-defined	143 (60.34)	54 (54.00)	
Lobulated or irregular	70 (29.54)	27 (27.00)	
Extrathyroidal extension	24 (10.13)	19 (19.00)	
Echogenic foci^b^			0.465
No calcification	68 (28.69)	20 (20.00)	
Macrocalcifications	63 (26.58)	23 (23.00)	
Peripheral calcifications	6 (2.53)	2 (2.00)	
Microcalcifications	147 (62.03)	72 (72.00)	
TI-RADS classification^b^			1.000
III	1 (0.42)	0 (0)	
IV	19 (8.02)	8 (8.00)	
V	216 (91.14)	92 (92.00)	
ETE			0.910
Negative	165 (69.62)	69 (69.00)	
Positive	72 (30.38)	31 (31.00)	

Continuous variables were presented with Mean ± SD and were calculated by Mann-Whitney U test. Data marked with ^a^ were calculated by Student's t-test. Categorical variables were presented with number and percentage (percentage in parentheses) and were calculated by Chi-square analysis. Data marked with ^b^ were calculated by Fisher exact test.

ALB = albumin; ALT = alamine aminotransferase; ANTITGAB = anti-thyroglobulin antibodies; ANTITPOAB = anti-thyroid peroxidase antibody; AST = asparate aminotransferase; BUN = blood urea nitrogen; Ca = calcium ion; CREA = creatinine; ETE = extrathyroidal extension; FT3 = free triiodothyronine 3; FT4 = free triiodothyronine 4; HB = hemoglobin; LYM = lymphocyte; NEUT = neutrophil; PLT = platelets; RBC = red blood cell; SD = standard deviation; TG = thyroglobulin; TI-RADS = Thyroid Imaging Reporting and Data System; TSH = thyroid stimulating hormone; TT3 = total triiodothyronine 3; TT4 = total triiodothyronine 4; UA = uric acid; URBC = urinary red blood cell; WBC = white blood cell

### Sonographic method for extrathyroidal extension predicting

**Figure 2.**
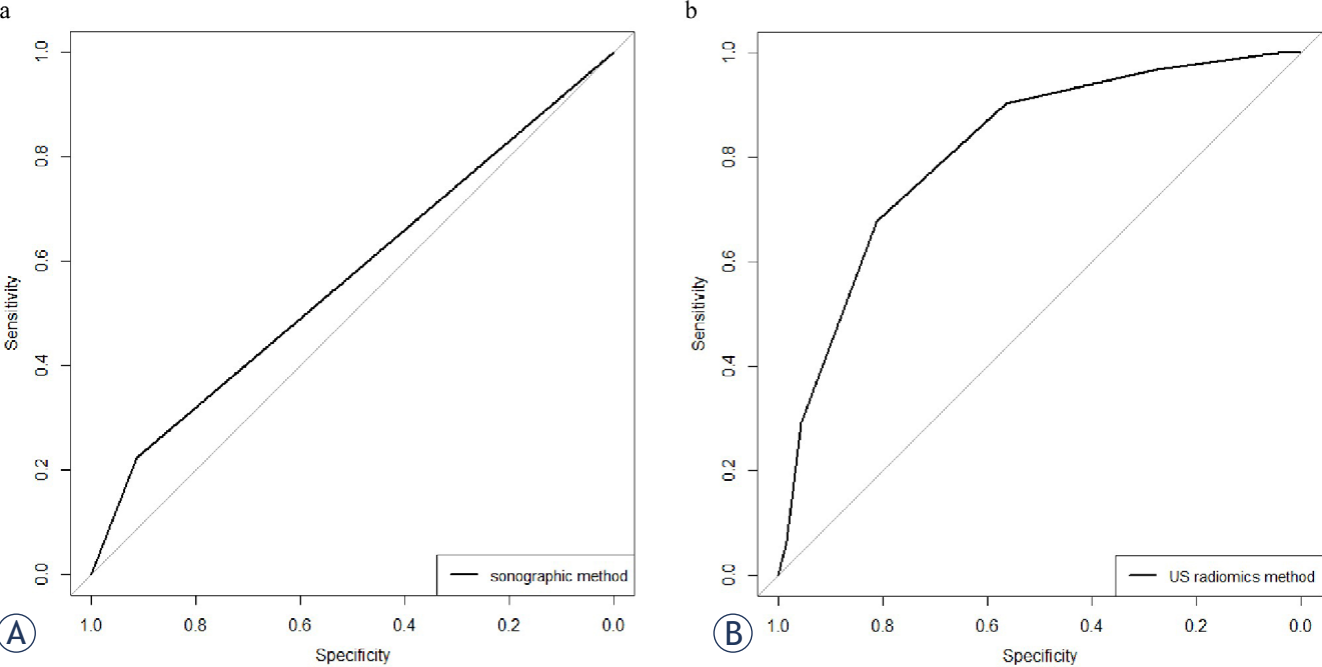
The ROC curves of sonographic method and US radiomics method for ETE predicting. **(A)** The ROC curve of sonographic method in all nodules. **(B)** The ROC curve of US radiomics method in validation group. ETE = extrathyroidal extension; ROC = the receiver operating characteristic; US = ultrasonic

The predictive performance for sonographic method was summarized in Table 2. And its ROC was shown in Figure 2A. A relatively high accuracy (0.70), specificity (0.7279) and NPV (0.9145) was found for this method. However, its sensitivity (0.5349), PPV (0.2233) and AUC (0.569) was at a low level.

**Table 2.** Comparison of predictive performance for models and sonographic method for extrathyroidal extension predicting

**Model**	**Accuracy (95% CI)**	**Sensitivity**	**Specificity**	**PPV**	**NPV**	**AUC**	***p*-value**
xgboost	0.77(0.6751–0.8483)	0.6774	0.8116	0.6176	0.8485	0.813	-
RF	0.73(0.6320–0.8139)	0.3548	0.8986	0.6111	0.7561	0.741	0.000006
GBM	0.75(0.6534–0.8312)	0.5164	0.8551	0.6154	0.7973	0.737	0.000012
binary LR	0.74(0.6427–0.8226)	0.6774	0.7681	0.5676	0.8413	0.730	0.000237
NB	0.55(0.4473–0.6497)	0.9355	0.3768	0.4028	0.9286	0.656	0.000000
DT	0.68(0.5792–0.7698)	0.3871	0.8116	0.4800	0.7467	0.634	0.000000
adaboost	0.71(0.6107–0.7964)	0.3548	0.8696	0.5500	0.7500	0.612	0.000000
SVM	0.70(0.6002–0.7876)	0.2903	0.8841	0.5294	0.7349	0.567	0.000000
KNN	0.69(0.5897–0.7787)	0.1935	0.9130	0.5000	0.7159	0.553	< 2.2x10^-16
Sonographic method	0.70(0.6514–0.7515)	0.5349	0.7279	0.2233	0.9145	0.569	< 2.2x10^-16

adaboost = adaptive boosting; AUC = area under the curve; CI = confidence interval; DT = decision tree; ETE = extrathyroidal extension; GBM = gradient boosting machine; KNN = k nearest neighbour; LR = logistics regression; NB = naivebayes; NPV = negative predictive value; PPV = positive predictive value; RF = randomforest; SVM = support vector machine; xgboost = the extreme gradient boosting

### US radiomics method for extrathyroidal extension predicting

After standardization, the top-99 most relevant features to ETE were retained using mRMR. And 81 candidate features were selected using LASSO algorithm afterwards. Among the nine predicting models, xgboost model had the highest accuracy (0.77) and AUC (0.813) with *p*-value <0.05 compared to other models (Table 2). Meanwhile, with its relatively high specificity (0.8116), and NPV (0.8485), it was chosen as US radiomics method. Then the predictive performance of US radiomics method and sonographic method was compared. And the results showed that the AUC of US radiomics method was significantly higher than that of sonographic method with *p*-value <0.05. Meanwhile, the accuracy, sensitivity, specificity and PPV of US radiomics method was higher than that of sonographic method (Figure 2B, Table 2). Thus, we believe US radiomics method surpassed sonographic method in ETE predicting (Figure 1).

### Partial dependence profile of US radiomics method

**Figure 3.**
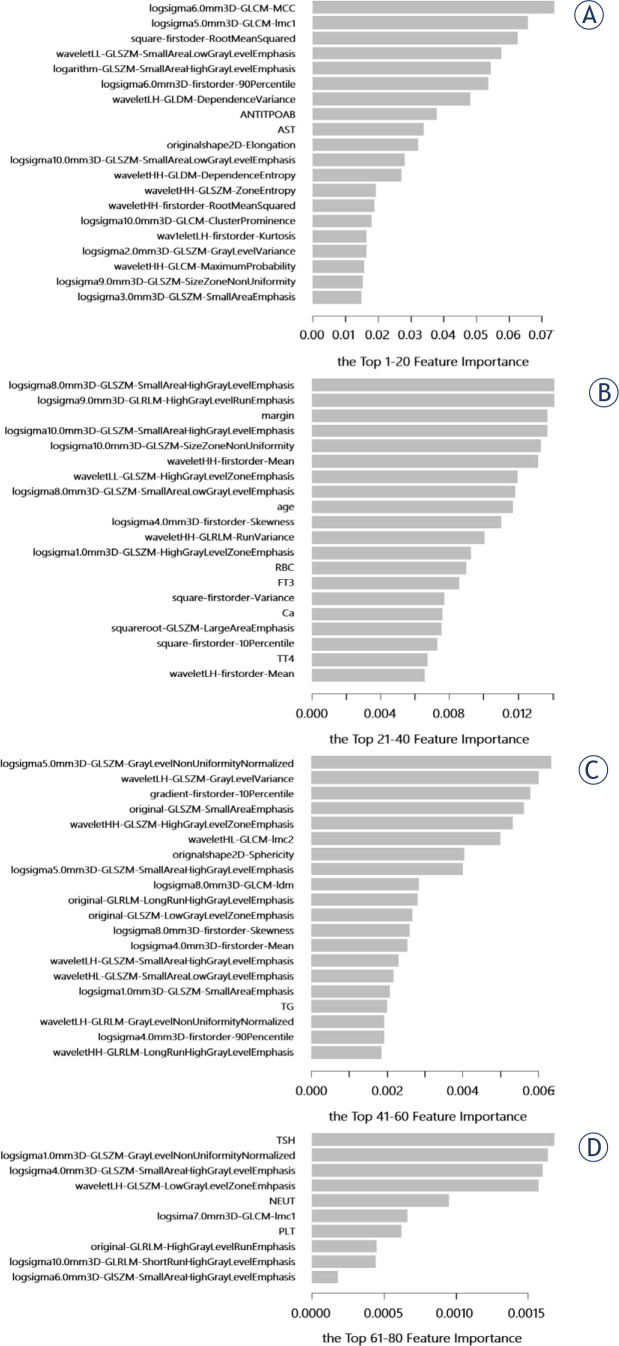
The image of feature importance for xgboost model. **(A-D)** the top 1–20, top 21–40, top 41–60 and top 61–80 feature importance of xgboost model. xgboost=the extreme gradient boosting; GLSZM=Gray-level size-zone matrix; GLCM=gray-level co-occurrence matrix; GLDM=gray-level dependence matrix; GLRLM=gray-level runlength matrix; LH=low-pass/high-pass; LL=low-pass/low-pass; HH=high-pass/high-pass; HL=high-pass/low-pass; ANTITPOAB=anti-thyroid peroxidase antibody; AST=asparate aminotransferase; RBC=red blood cell; FT3=free triiodothyronine 3; Ca=calcium ion; TT4=total triiodothyronine 4; TG=thyroglobulin; TSH=thyroid stimulating hormone; NEUT=neutrophil; PLT=platelets.

**Figure 4.**
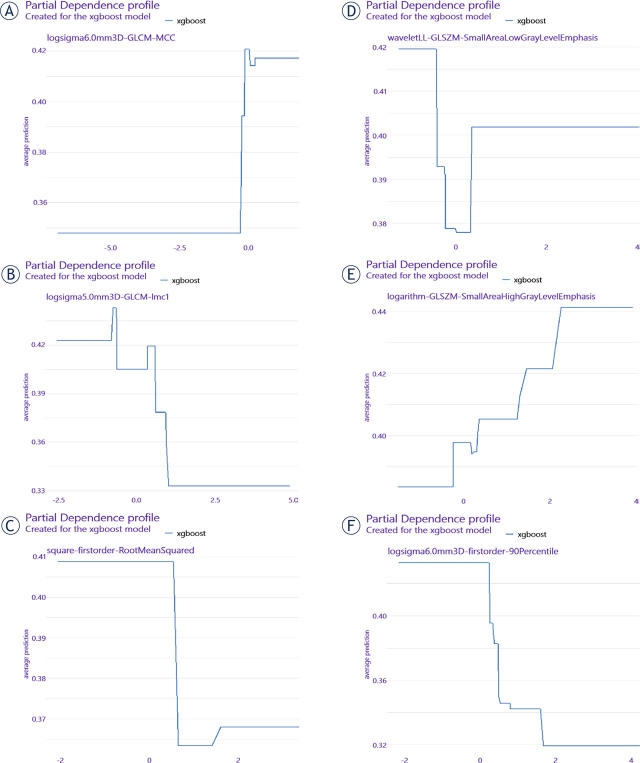
Partial dependence profile of the top-6 important features in xgboost model. **(A-F)** Partial dependence profile of logsigma6.0mm3D-GLCM-MCC, logsigma5.0mm3D-GLCM-Imc1, square-firstorder-RootMeanSquared, waveletLL-GLSZM-SmallAreaLowGrayLevelEmphasis, logarithm-GLSZM-SmallAreaHighGrayLevelEmphasis and logsigma6.0mm3D-firstorder-90Percentile in xgboost model in training group. xgboost, the extreme gradient boosting; GLSZM, Gray-level size-zone matrix; GLCM, gray-level co-occurrence matrix; LL, low-pass/low-pass.

The image of feature importance for xgboost model was shown in Figure 3. And the top-6 features were chosen at a cut-off of 0.05 (Gain value). The partial dependence profiles of the them were revealed in Figure 4, in which the variation trends between features and ETE states were presented.

## Discussion

The association between minimal ETE and poor prognosis of PTC patients had been questioned since 2006.^22^ In 2017, the AJCC tumor-node-metastasis staging system changed the stratification criteria and excluded minimal ETE as an isolated risk factor for poor prognosis.^9,22^ However, controversial opinions and contradictory results arose consistently. In resent review of SEER database, which contained approximately 10% of differentiated thyroid cancer patients in America, Liu *et al.* found that patients with minimal ETE had significantly lower rates of cancer-specific survival and overall survival than those without.^3^ Danilovic *et al.* revealed 596 PTC patients and indicated that both minimal ETE and gross ETE were independent risk factors of recurrence, although gross ETE might lead to a worse one.^23^ What's more, Almeida *et al*. concluded that minimal ETE was the only aggressive feature for low-risk PTC after analyzing over 1100 PTCs.^22^ In clinical practice, the detection of ETE determined the selection of optimal treatment of PTC patients. Usually, total thyroidectomy or subtotal thyroidectomy was suggested for patients with ETE while hemithyroidectomy was recommended for those without. However, surgical procedure of hemithyroidectomy didn't only retain some functionality of the thyroid but also protect parathyroid functions and contralateral laryngeal recurrent nerve.^7^ Therefore, an effective way to evaluate ETE preoperatively could help patients in multi-aspect.

In this study, we retrospectively analyzed 337 PTC nodules, which included 4 gross ETE and 99 minimal ETE, and measured the predicting value of sonographic method and US radiomics method. The sensitivity, specificity, PPV, NPV, accuracy and AUC of sonographic method accounted for 53.49%, 72.79%, 22.33%, 91.45%, 70% and 0.569. Those values of US radiomics method accounted for 67.74%, 81.16%, 61.76%, 84.85%, 77% and 0.813. Thus, we concluded that the predicting value of US radiomics method surpassed sonographic method. Meanwhile, we found the top-6 important features selected by xgboost model included two GLCM, two GLSZM and two first-order features, among which four LOG (sigma values: 5.0 mm and 6.0 mm), one wavelet (LL), one square and one logarithm filters were acquired.

In studies of Lamartina, Han, Hu and Lee, the sensitivity, specificity, PPV, NPV, and accuracy of sonographic assessment for minimal ETE or overall ETE varied from 30.0% to 79.7%, from 79.5% to 93%, from 51.0% to 76.1%, from 76.6% to 86.1%, and from 71.7% to 81.9%, respectively.^10,24,25,26^ Consistent to our study, the sensitivities of their results were lower than the specificities. Similar trend was found in PPVs and NPVs. However, Han reported an AUC of 0.746 for ETE predicting, which was higher than our result.^24^ That might be explained by the smaller population size (n = 111) acquired in their study.^24^ To our knowledge, only one article about US radiomics prediction of ETE was published by Wang in 2021.^7^ In their study, they included clinical characteristics like age, sex, size and radiological ETE diagnosis, as well as location, border and vascularization elastic properties classification of tumor. The radiomic features they extracted included first-order features, shape features and texture features. And the texture features covered GLRLM, GLSZM, GLDM, GLCM and neighbourhood grey-tone dependency matrix (NGTDM). A radiomic nomogram model was selected as the best model with an AUC of 0.824 in validation group. It obtained two clinical characteristics (tumor location and radiologist diagnosis of ETE) and one radiomics signature, which was comprised of waveletLL-GLSZM, NGTDM, waveletHH-GLCM, waveletLH-GLCM corr, waveletLH-GLCM clus and waveletLH-GLSZM. Similar to their study, clinical characteristics we acquired were less important than radiomic features, and the majority of selected radiomic features were GLCM and GLSZM features.

First-order features mainly described the distribution of voxel intensities while texture features measured the inter-relationship between voxel distributions.^27^ GLCM represented the grey levels of neighbouring pixels with spatial relationship in an image and was commonly applied in studies.^28^ The direction independent technique, GLSZM, quantified homogeneous zones for specific grey level in an image.^28^ LOG filter was a combination of Laplacian operator and Gaussian filter, which could decrease the noise and the impact of signals in a medical image.^29,30^ What's more, it could detect edges and smooth images. With different sigma parameters, a series of textural coarseness were derived for further evaluation.^30^ Another widely used filter, wavelet, focused on individual voxels and the relationship between them.^31^ It could enhance certain characteristics of images with decomposition of high-pass and low-pass in the x and y directions.^31^ In our study, we found that all the important features we selected in xgboost model were high-dimensional features. Besides, we found a higher rate of LOG filtered features than other filters. Taken together with different functions of filters, we concluded that the LOG filter might had better predicting value than others in our study.

Preoperative US examination was the first-line diagnostic tool for PTC patients to detect the presence of lymph node metastasis and ETE.^10^ However, it had the disadvantage of subjectivity and reliance on the experience level of operator.^7^ US radiomics could solve the problems for it extracted and recognized the voxel intensities and distributions automatically whereas accurately predicted ETE preoperatively. Thus, it could help PTC patients from avoiding unnecessary operations and reducing the risk of reoperation.^7^

The limitations in our study were summarized as following. First of all, the clinical procedure was not strict due to the retrospective nature. With the lack of clinical information or US images, several cases had to be excluded as a result. Secondly, capture of unrepresentative portion of tumors might happen because of discrete images. And that might increase the variability of US images and influence the repeatability of this study. Thus, we checked and chose images with strict standard to keep the image quality consistent. What's more, the conclusion was derived from relatively small sample in monocentric database. And larger cohorts in multicenter database were required in the future to improve its repeatability.

In conclusion, our study proved the superior value of US radiomics method to sonographic method in preoperatively detecting ETE for PTC patients. Meanwhile, we found high-dimensional radiomic features had better predicting value than clinical characteristics.
